# Assessment of inpatient antibiotic use in Halibet National Referral Hospital using WHO indicators: a retrospective study

**DOI:** 10.1186/s13104-018-4000-7

**Published:** 2018-12-18

**Authors:** Nebyu Daniel Amaha, Yohana Haile Berhe, Atul Kaushik

**Affiliations:** 1 School of Pharmacy, Asmara College of Health Sciences, 118-Adi Shimagle, P.O. Box 8566, Asmara, Eritrea; 2 Pharmacy, Halibet National Referral Hospital, Asmara, Eritrea

**Keywords:** Antibiotic, Rational drug use, WHO indicators, Inpatient study, Prescription

## Abstract

**Objective:**

Inappropriate use of antibiotics in primary care and hospital settings is a major contributing factor to the spread of antibiotic resistance. Many microorganisms were tested in Eritrea and have proven resistant to ampicillin. The aim of this study was to investigate the prescription pattern, hospital indicator and patient care indicator of antibiotics among hospitalized patients in Halibet National Referral Hospital, Asmara, Eritrea.

**Results:**

The data on prescription patterns showed 79% of hospitalizations had at least one antibiotic prescribed and on average 1.29 antibiotics were prescribed per hospitalization; prescribing using generic name was at 97%; all (100%) of the antibiotics were prescribed from the Eritrean National List of Medicines. On average an antibiotic was prescribed for 6.36 days (SD = 6.06). Ampicillin was the most commonly prescribed antibiotic (42.1%) and parenteral was the most common route prescribed (81.4%). The data on hospital indicators showed key antibiotics were out of stock on average for 78.18 days; 87.5% of key antibiotics were available on the day of the study. The data on patient care indicator showed patients taking antibiotics stayed in the hospital for 9.97 days (± 7.33 days).

**Electronic supplementary material:**

The online version of this article (10.1186/s13104-018-4000-7) contains supplementary material, which is available to authorized users.

## Introduction

Irrational use of antibiotics in inpatient settings is an important issue that needs attention. Some causes for inappropriate drug use in hospitalized patients are; unnecessary use of antibiotic, inadequate regulation and monitoring, inappropriate dose, and multiple antibiotic prescribing [1–4]. Lack of Standard Treatment Guideline (STGs), protocols for rational drug use, and weak or absent Drug Therapeutic Committees (DTCs) exacerbate the inappropriate antibiotic use [5]. About 70% of hospital-acquired bacterial infections are resistant to at least one antibiotic [6].

WHO has developed key indicators to measure antibiotic use in health facilities [7] and many studies [8–16] have reported using the WHO indicators. These indicators also known as “core drug use indicators” include prescribing, hospital and patient-care indicators were developed to be used at primary healthcare facilities and later adapted to tertiary hospitals [4] (Additional file 1: Annex S3). However, these indicators do not address factors like duration of hospital stay or the type of disease being treated which could influence antibiotic use [17]. Thus the outpatient focused WHO core drug use indicators were modified to the inpatient settings.

High rate of antibiotic use has been reported in Eritrea [18], however, published research on antibiotic use in Eritrean hospitals is meager [19]. The hospital indicators focus on studying the availability and stock outs of key antibiotics. A key antibiotic for the purposes of this study is a drug having a “V” (vital drug) designation in the ENLM and that is used at a hospital level. Vital medicines are defined as life-saving, crucial medicines which are of the highest therapeutic importance for the provision of basic health service [20].

A recent study [21] in Orotta national referral Hospital in Asmara found that most of the bacterial isolates tested were resistant to ampicillin, ceftazidime, nitrofurantoin, co-trimoxazole, nalidixic acid and tetracycline. According to this study the percentage of resistance to ampicillin in *E. coli* was (87.5%), *Klebsiella* spp (75%), *Citrobacter* (100%), *Pseudomonas* (81.8%) and *Salmonella* (100%) [21]. Another study conducted on the antimicrobial susceptibility of *S. aureus* in Eritrea found that 85% were resistant to ampicillin [22].

The aim of this study was to investigate the antibiotic use in hospitalized patients in Halibet National Referral Hospital (HNRH) using an indicator based methodology designed by Strengthening Pharmaceutical Systems (SPS) [17].

## Main text

### Methods

#### Setting

The study was conducted at HNRH, one of the two national referral hospitals in Asmara, Eritrea. HNRH is a 180-bed national referral and teaching hospital with Surgical, Medical, Orthopedic, Burn and Emergency wards.

#### Study design

A cross-sectional, retrospective study design was used to evaluate three key drug use indicators viz. hospital indicators, prescribing indicators and patient care indicator (Additional file 1: Annex S3). The methods for collecting and evaluating these indicators were adopted from the Strengthening Pharmaceutical Systems Program [17] after studying the appropriateness of the indicators to the hospital environment (see Additional file 1: Annex S1).

#### Eligibility criteria

Patients admitted from January 1, 2017, until December 31, 2017, and had their medication dispensed from the inpatient pharmacy, irrespective of their age, gender and diagnosis were included.

#### Sample size and data collection

A clinical card list of 3654 patients was prepared according to their admission dates, those admitted in January coming first and those admitted in December appearing last. We randomly picked one number (X) from 0 to 9, then by adding 300 to the number, a second number (X + 300) was obtained. Starting from the second number every 300th card number was included to select 100 cases from the 3654 cases. The number of prescribing encounters to be studied were chosen based on the WHO’s 1993 recommendations [7]. The complete patient chart for these samples was obtained from the record office with prior permission of the Medical Director of the hospital. The data collection forms were slightly modified and the applicable data was collected for a month i.e. March to April 2018 using pre-defined data collection forms (see Additional file 1: Annex S1).

#### Statistical analysis

After checking for data completion, the necessary computations were performed according to the formulas given (Additional file 1: Annex S2). Statistical analysis such as frequencies, percentage and averages were carried out using Statistical Package for Social Sciences (IBM, SPSS Statistics for Windows, version 20. Armonk, NY: IBM Corp.).

### Results

#### Prescribing indicators

The study found that 79% of the patients received at least one antibiotic. On average 1.29 antibiotics were prescribed per hospitalization and all (100%) were prescribed from the EML while 97% of the antibiotics were prescribed using generic names. On average patients were treated with an antibiotic for 6.36 days (Table 1).

**Table 1:** Comparison of inpatient drug use indicators in HNRH with WHO ideal values

Core drug use and In-patient antibiotic use indicator	Result (SD)	WHO [13, 17]
Percent of hospitalization with one or more antibiotics prescribed	79%	20–26%^a^
Average number of antibiotics prescribed per hospitalizations	1.29	1.6–1.8^b^
Percent of antibiotics from National or Hospital Formulary	100%	100%
Average number of days antibiotic treatment was prescribed	6.36 (6.06)	–
Percent of antibiotics prescribed by generic name	97%	100%
Percentage of key antibiotics available on the day of study	87.5%	100%
Average number of days that a set of key antibiotic was out of stock	78.18	–
Expenditure on antimicrobials as a percentage of total hospital medicine cost	25.59%	20–40
Average number of days of patients who received antibiotics stayed in hospital	9.97(7.33)	–
Percent of antibiotics prescribed in injection form	81.4%	13.4–24.1^a^

*SD* standard deviation

^a^WHO optimal value for outpatient departments

^b^WHO indicator for all medicines (including antibiotics) prescribed per encounter

#### Hospital indicators

The hospital lacks a Standard Treatment Guideline and a hospital formulary. On the day of the study 87.5% of the antibiotics were available in stock and HNRH spends 25.59% of its medicines budget on antimicrobials. A key antibiotic was out of stock for 78.18 days in 2017 (Table 1).

#### Patient care indicator

Patients taking antibiotics, on average, stayed for 9.97 days in the hospital, with the standard deviation of 7.33 days (Table 1). Intravenous injection was the most common route of administration (81.4%) and 77.2% of patients were given an antibiotic for less than 7 days. Ampicillin was the most commonly prescribed antibiotic and 77% of patients received only one antibiotic while 17% were treated with two antibiotics simultaneously and 6% were being treated with three antibiotics at the same time.

#### Auxiliary indicators

Ampicillin was the most commonly prescribed antibiotic in HNRH (Table 2). Most of the patients (77%) had only one antibiotic prescribed (Table 2). We found 77.2% patients took antibiotics 1 to 7 days (Table 2). Around quarter (26%) of the patients stayed from 10 to 20 days, while 6.5% stayed longer than 21 days (Table 2).

**Table 2:** Route of administration, number per hospitalization, treatment days, most commonly prescribed antibiotics and hospital stay days in HNRH

Antibiotic treatment days	1–7 days	77.2%
	7–14 days	13.9%
	15–20 days	2.53%
	> 21 days	3.79%
Number of antibiotics per hospitalization	One antibiotic	77%
	Two antibiotics	17%
	Three antibiotics	6%
Most commonly prescribed antibiotics	Ampicillin	42.1%
	Benzyl penicillin	13.7%
	Gentamycin	9.8%
	Cloxacillin	8.8%
	Ceftriaxone	5.9%
	Ciprofloxacin	4.9%
Days spent in the Hospital	1–10 days	67.5%
	11–20 days	26%
	> 21 days	6.5%

### Discussion

#### Prescribing indicators

We found 79% of the admitted patients had received at least one antibiotic during their hospital stay (Table 1). This figure is higher than the 73.7% found by a specialty-hospital based study in Ethiopia (Table 3). Other ward-specific hospital studies reported lower antibiotic use percentages, for instance, in Ethiopia (64.7%) [23], (73.68%) [24] and 66% in India [25]. A point-prevalence antibiotic use study in 11 hospitals in the Democratic Republic of Congo reported 68% of patients had received an antibiotic during their hospital stay [26] (Table 3). A study focusing on the surgical ward of HNRH in 2009 found antibiotic use prevalence to be 69%, with 30% used preoperatively and 39% postoperatively [27].

**Table 3:** Comparison of antibiotics use with other African countries

Indicator		Ethiopia [8, 16, 23, 28, 34]	Sudan [35]	DR Congo [26]	Zambia [36]
Percent of hospitalization with one or more antibiotics prescribed	79%	73.7%	81.3%	68%	53.7%^a^
Average number of antibiotics prescribed per hospitalizations	1.29	2.1	2^b^	1.4	2.5^b^
Generic name prescribing	97%	90.6%	49.3%	NS	56.1%
Most commonly prescribed antibiotic	Ampicillin	Ceftriaxone	NS^c^	Ampicillin	Amoxicillin^a^
Parenteral route of administering antibiotics	81%	82.4%	3.5%^a^	68.2%	11.8%^a^
Percentage of patients taking two or more antibiotics	23%	65%	NS	34.9%	
Availability of key antibiotics	87.5%	65.7%	81.3%	NS	83.3%
Average days of antibiotic stock out per year	78.18	30	NS	NS	
Drugs prescribed from EML	100%	96.6%	57.2%	NS	98.1%

^a^Outpatient department prescriptions

^b^Number of all drugs prescribed including antibiotics

^c^Data not stated

This study showed that on average 1.29 antibiotics were prescribed per hospitalization (Table 1), lower than a study in the DRC in which 1.4 antibiotics were prescribed per patient [26] and much lower than the 2.1 antibiotics per patient reported in an Ethiopian hospital [28] (Table 3).

All (100%) of the prescribed antibiotics were in the National Essential Medicines List (EML). This is because HNRH procures almost all of its medicines from one supplier, PHARMECORE, which only procures according to the EML. Therefore prescribers in HNRH are encouraged to adhere to the EML, resulting in 100% adherence to the EML. Numerous studies reported varying percentages of EML adherence; Lesotho (79%) [29], Pakistan (98.8%) [4], and India (99.8%) [9].

In HNRH an antibiotic was prescribed on average for 6.36 days (SD = 6.06) (Table 1). However 13.1% of patients were prescribed antibiotics longer than 10 days while 7% stayed on antibiotics longer than 14 days and 3% of patients were given an antibiotic longer than 20 days.

Overprescribing injectable medicine is considered a case of inappropriate antibiotic use [4, 30]. Parenteral was the most common route of administration consisting 81.4% of all prescribed antibiotics. This is comparable to a study in Ethiopia (82.4%) [28] but quite higher than in the DRC (68.2%) [26] (Table 3). Thus prescribers in HNRH need to consider switching to oral route of administration which is associated with lower treatment cost, catheter-related infections, hospital stay and burden for nursing staff [31].

#### Hospital care indicators

On the day of the study 87.5% of the key antibiotics were available in stock in the stores of HNRH. Similar studies done in developing countries found key antibiotics were available 72.4% in Pakistan [4] and 65.7% in Ethiopia [16] (Table 3). Our finding although better than what has been reported from Pakistan [4] and Ethiopia [16], it is less than ideal because key drugs should be available at all times [20]. Lack of access to key antibiotics forces prescribers to make less appropriate drug choices with higher costs and more risk of side effects and antibiotic resistance emergence. In HNRH key antibiotics were out of stock for an average of 78.18 days per year, much higher than the 30 days reported in Ethiopia [8] (Table 3).

#### Patient care indicators

The average number of hospital stay for patients in HNRH was 9.97 days (Table 1). This is much higher than the average 6 days reported by a study in a private hospital in India [25]. Furthermore, our finding shows 32.5% of the patients stayed longer than 10 days. Staying longer than 10 days is 3.086 times more likely to result in antibiotic use problems than when staying less than 10 days [31]. Prolonged hospital stay is also associated with higher treatment costs, increased risk of nosocomial infections, the emergence of resistant microorganisms and increased risk of ADR and drug–drug interaction [17, 32, 33]. We found that 6.5% of the patients in HNRH stayed longer than 20 days which could result in increased risk of antibiotic use problems. Unnecessarily longer duration of antibiotic use in HNRH needs to be addressed by developing an STG and closer therapeutic monitoring of patients taking systemic antibiotics.

## Limitations of the study

The limitations which need to be considered are as follows. Firstly, the findings of this study could not be generalized to the whole of Eritrea since this was done in a single hospital. Secondly, this study was done in the inpatient department of the hospital and thus it does not reflect the outpatient prescribing patterns of the hospital.

## Additional file

**Additional file 1: Annex S1.** Data collection forms. **Annex S2.** Formulas used to calculate the indicators. **Annex S3.** List of the three types of drug use indicators investigated in HNRH. **Annex S4.** Medical Director’s ethical clearance.

## Abbreviations

CDC: Center for Disease Control
HNRH: Halibet National Referral Hospital
DRC: Democratic Republic of Congo
DTC: Drug and Therapeutic Committee
EML: Essential Medicines List
ENLM: Eritrean National List of Medicines
I.V.: Intravenous
MoH: Ministry of Health
SPS: Strengthening Pharmaceutical Services
SPSS: Statistical Package for Social Sciences
STG: Standard Treatment Guideline
WHO: World Health Organization
