# Supplementary material for: Assessment of inpatient antibiotic use in Halibet National Referral Hospital using WHO indicators: a retrospective study
Source: BMC Res Notes. 2018 Dec 18;11:904. doi: 10.1186/s13104-018-4000-7 (PMC6299551; doi:10.1186/s13104-018-4000-7)
Supplement: Supplementary file 1 — Additional file 1: Annex S1. Data collection forms. Annex S2. Formulas used to calculate the indicators. Annex S3. List of the three types of drug use indicators investigated in HNRH. Annex S4. Medical Director’s ethical clearance. [file 13104_2018_4000_MOESM1_ESM.docx]

**ANNEX S1. BLANK DATA COLLECTION FORMS**

***How to Investigate Antimicrobial Use in Hospitals: Selected Indicators***

***Instrument 1***

**Form to Record Antimicrobial Treatments**

Name of unit: _______________________________ Data collector: __________________________ Date: ______________

**Patient Information Antimicrobial Information**

**1 2 3 4 5 6 7 8 9 10 11 12 13 14 15 16 17**

Total

Cases

Total

*Y*s Total *Y*s

Total

Days Total *Y*s Total

Total

*Y*s

Total

*Y*s ___

Total

Days

Total

Generics ___

Total

Doses

Total

Doses ___ Total Cost

Total

Cost

[Instrument 2 is used to collect information for Indicators 6, 7, 8, 9, 10, 13, 14, 15, 16, and 17.]

*Notes:* No. = number, Y = Yes, N = No, INN = international nonproprietary name, FL = formulary list, * = TB treatment provided by central Ministry of Health program

***Annex S1. Blank Data Collection Forms***

***Instrument 2***

**Availabilit*y* of a Set of Ke*y* Antimicrobials and Time Out of Stock**

Name of unit:___________________ Data collector:_________________ Date:______

**1 2 3 4 5 6 7 8 9 10 11 12 13 14 15**

**Product**

**(Generic Name,**

**Current**

**M1**

**Last**

**Days Out of Stock**

**Total**

**Days Out**

**Form, and Strength)**

**Stock**

**month M2 M3 M4 M5 M6 M7 M8 M9 M10 M11 M12**

**of Stock**

**Total:**

[Instrument to collect information for Indicators 3 and 4.]

**Instructions:**

1. Review the stock sheet for each essential or referenced antimicrobial. Record the current stock quantity in column 2 and then, for each month, record the number of days for which the product was out of stock, starting with last month and working backward.

2. Add the number of days out of stock for each month and enter the total in the last column.

**Calculations:**

**Indicator 3** = Add the total numbers of entries in column 2 that are more than 0 and divide by the number of products in column 1.

**Indicator 4** = Add the total numbers of entries in column 15 and divide by the number of products in column 1

**ANNEX S2. FORMULAS**

**Formulas used to calculate the indicators**

**Prescribing indicators**

1. $Percentage of hospitalizations with one or more antibiotics prescribed=\frac{Number of patient hospitalizations with one or more antibiotics prescribed}{Total number of hospitalizations studied}x 100$
2. $Average no. of antibiotics prescribed pe rhospitalization in which antibiotics were prescribed= \frac{Number of antibiotics prescribed for all hospitalizations}{Total number of hospitalizations with antibiotics prescribed}$
3. $Percentage of antibiotics prescribed consistent with the hospital formulary list=\frac{Number of antibiotics prescribed that are on the formulary list}{Number of antibiotics prescribed}x 100$
4. $Average duration of prescribed antibiotic treatment=\frac{Total number of days on antibiotic treatment}{Total number of antibiotics prescribed}$
5. $Percentage of antibiotics prescribed by generic name = \frac{Total number of antibiotics prescribed by generic name}{Total number of antibiotics prescribed}x 100$

**Hospital indicators**

1. $Percent of key antibiotics available on study day =\frac{Number of key antibiotics actually in stock}{Number of key antibiotics that should be available}x 100$
2. $Average number of days that a set of key antibiotics is out of stock =\frac{Number of days that each key antibiotic is out of stock}{Number of key antibiotics in the review}$
3. $Expenditure on antibiotics as a percentage of total hospital medicine costs =\frac{Total cost of all antibiotics purchased}{Total cost of all medicines purchased}x 100$

**Patient care indicator**

1. $Average duration of hospital stay of patients who receive antibiotic =\frac{Total number of days of hospitalization for patients receiving antibiotics}{Number of patients receiving antibiotics}$

**ANNEX S3. LIST OF INDICATORS**

**List of the three types of drug use indicators investigated in HNRH**

**Prescribing indicators**

Percentage of hospitalizations with one or more antimicrobials prescribed

Average number of antimicrobials prescribed per hospitalization in which antimicrobials were prescribed

Percentage of antimicrobials prescribed consistent with the hospital formulary list

Average duration of prescribed antimicrobial treatment

Percentage of antimicrobials prescribed by generic name

Number of antibiotics per hospitalization

Antibiotic route of administration

Most commonly prescribed antibiotics

Distribution of antibiotic treatment days

**Hospital indicators**

Percent of key antimicrobials available on study day

Average number of days that a set of key antimicrobials is out of stock

Expenditure on antimicrobials as a percentage of total hospital medicine costs

**Patient care indicator**

Average duration of hospital stay of patients who receive antimicrobials

**ANNEX S4. MEDICAL DIRECTOR’S CLERANCE**

**
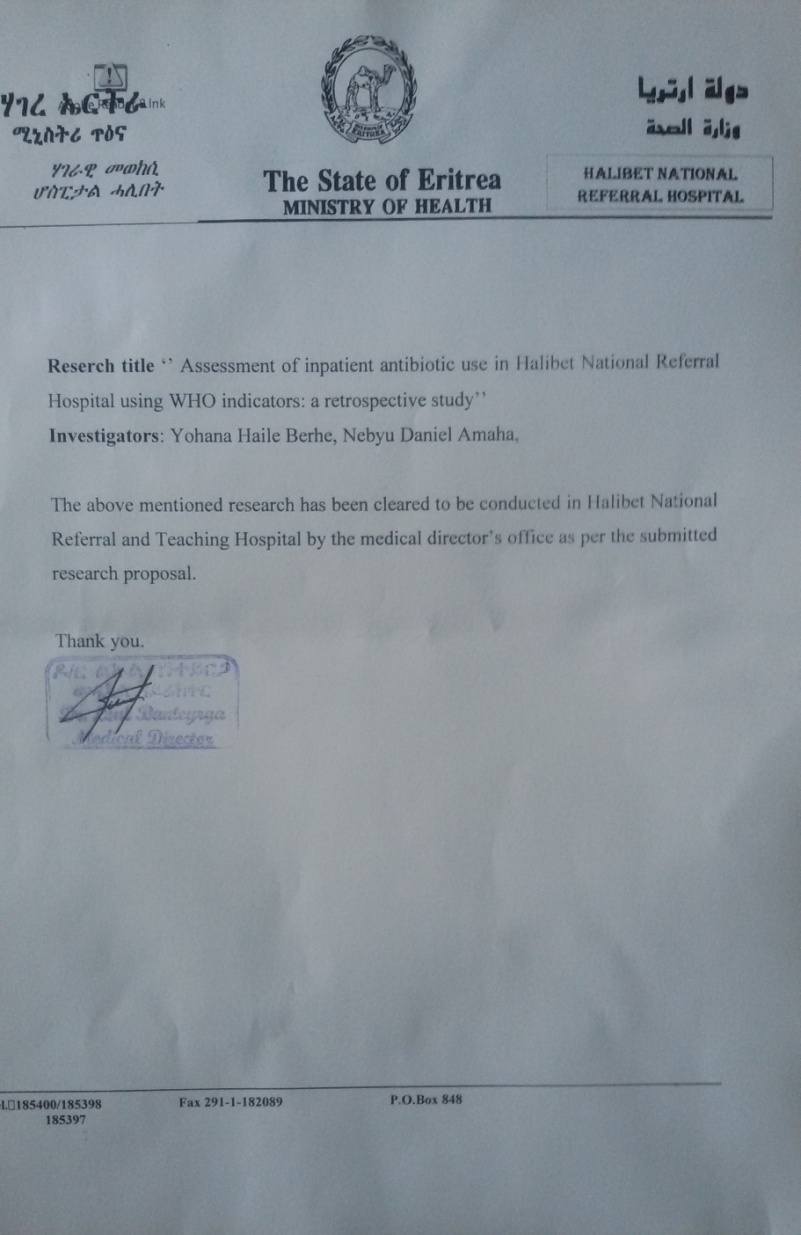
**
